# Can a linking crosswalk table be applied to a different population? An independent validation study for a crosswalk between BSI depression and PROMIS depression scales

**DOI:** 10.1371/journal.pone.0278232

**Published:** 2022-11-28

**Authors:** Xiaodan Tang, Benjamin D. Schalet, Patrick Janulis, Michele D. Kipke, Aaron Kaat, Brian Mustanski, Michael E. Newcomb, Amy Ragsdale, Soyeon Kim, Sue Siminski, Pamina M. Gorbach

**Affiliations:** 1 Department of Medical Social Sciences, Northwestern University Feinberg School of Medicine, Chicago, IL, United States of America; 2 Department of Epidemiology and Data Science, Amsterdam UMC, Vrije Universiteit, Amsterdam, The Netherlands; 3 Institute for Sexual and Gender Minority Health and Wellbeing, Northwestern University, Chicago, IL, United States of America; 4 Keck School of Medicine, University of Southern California, Los Angeles, CA, United States of America; 5 Fielding School of Public Health, University of California Los Angeles, Los Angeles, CA, United States of America; 6 Frontier Science Foundation, Boston, MA, United States of America; 7 Frontier Science Foundation, Amherst, NY, United States of America; 8 Department of Epidemiology, Fielding School of Public Health, University of California Los Angeles, Los Angeles, CA, United States of America; Yamaguchi University: Yamaguchi Daigaku, JAPAN

## Abstract

A linking procedure establishes a “bridge” between the scores from different patient-reported outcome (PRO) instruments that measure similar constructs. After developing a linking relationship however, it is critical to evaluate whether this relationship can be generalized to different groups. Our study aims to validate a published crosswalk for score conversion between the Brief Symptom Inventory Depression subscale and the Patient-Reported Outcomes Measurement Information System Depression 8a using an independent sample. Data were from a sample of young men who have sex with men (MSM), which differs in terms of participant age, race, and ethnicity from the sample used to develop the existing crosswalk. The validity of the newly derived crosswalk was evaluated in terms of the correlation, mean difference and standard deviation between the observed and the linked scores. The two crosswalks were further compared to evaluate if the difference was within an acceptable range. More than half of the item parameters obtained from the two samples were found to overlap in their confidence intervals. Differences between each pair of scores in the two crosswalks was within three T-score points, well within the range of each crosswalk score’s standard error. This study concludes that an existing crosswalk is replicable on a sample that differs from that used for crosswalk development, but future research should continue to examine the generalizability of the linked parameters and evaluate the reproducibility of this crosswalk to other populations.

## Introduction

Patient reports can play an important role in medical research and clinical care [1]. Patients provide different kinds of information on their health status, including mental and physical symptoms, treatment effects, and quality of life [2]. This information may be described as patient-reported outcomes (PROs), which are collected with patient-reported outcome measures (PROMs). PROMs allow researchers and clinicians to understand patients’ perspectives on their health directly and contrast with clinical reports–such as those based on physical, laboratory, or radiology tests–which require further interpretation by individuals other than the patient [3].

In the case of depressive symptom severity, a great number of PROMs have been developed over the years and many are currently in use [4]. These include the Patient-Reported Outcomes Measurement Information System (PROMIS)–Depression [5, 6], the Center for Epidemiologic Studies Depression Scale (CES-D) [7], the 9-item Patient Health Questionnaire (PHQ-9) [8], and the Brief Symptom Inventory Depression subscale (BSI) [9], among others. Depression PROMs may differ in content coverage, patient burden, administration mode, and length, each of which may attract different users for different reasons.

The use of multiple PROMs poses a problem, however, for the aggregation and interpretation of research findings from multiple outcome studies [10]. For example, researchers attempting to synthesize results on 19 adolescent depression trials had to contend with 10 different depression scales [11]. Another study recently identified aggregated PROM data from electronic health records as a potential rich source of “real world data” for researchers and regulators to generate knowledge [12]. This vision becomes more challenging to realize when multiple different PROs are used across clinical settings.

Researchers can tackle this challenge by applying psychometric methods to harmonize data from multiple PROMs, including depression. For example, adopting techniques based on item response theory (IRT) and educational testing [13], PRO researchers have applied the methodology of *linking* (also known as *scale alignment* or *equating*) to PROMs scores by translating scores from multiple instruments to a common metric [14–16]. After the linking relationship between the scores of different PROMs is established, a score cross-walk table can be used to enable a one-to-one score conversion between PROMs [17, 18]. This crosswalk can contribute to subsequent research and clinical practice in terms of aggregating and comparing patient-reported outcomes collected with different instruments.

Measurement invariance is a psychometric property of an instrument, which demonstrates that measurement properties do not vary across populations. To evaluate measurement invariance, researchers need to examine whether an instrument performs the same way with different groups. Many researchers have examined measurement invariance of PROMs. For example, several studies have examined the performance of the PHQ-9 [19], the Beck Depression Inventory [20, 21], the Occupational Depression Inventory [22], 12-Item Short Form Survey (SF-12) [23] between different populations and between different assessment types [24]. Similarly, a successful linking relationship is assumed to meet the group invariance property, which states that the linking relationship between two instruments is the same regardless of the sample characteristics, such as differences in gender, race, ethnicity, age [13, 17, 25]. However, this property needs to be verified in practice, as it may not hold under certain theoretical conditions of linking methods [13, 25]. Lord and Wingersky (1984) pointed out that although the results of true-score linking methods–such as those based in item response theory (IRT)–can be subpopulation invariant, true scores were not theoretically justified as equal to observed scores [26, 27]. For this reason, the group invariance assumption should be investigated for each linking analysis. Several linking studies [16, 28–31] have computed the standardized root-mean-square deviation (RMSD) to evaluate subpopulation invariance [25] to determine if the linking relationship performs differently in diverse samples. However, this calculation is limited to the subpopulation groups existing in the analyzed dataset. As a practical matter, group invariance is difficult to examine exhaustively based on this approach, if only because of the absence of appropriate data.

Considering that group invariance cannot be strictly assumed to exist for a given linking analysis, researchers must qualify that the linking results only apply to samples from the same population used in the linking analysis [13]. PRO linking studies are often based on data from a single patient or general population sample [16, 28, 30, 32, 33]. As stated above, the linking results may be variable across different samples [17]; therefore, to better facilitate comparative clinical research and practice in terms of generalizability, linking researchers have recommended validation of linking crosswalks in multiple samples [15, 16, 31, 34]. Hence, it is important to investigate if the linking of two PROMs derived from one patient group can be applicable to another group.

### The current study

Our study examines whether a depression linking relationship established in one patient group is valid for use in another. Our study follows the data harmonization aims of the Collaborating Consortium of Cohorts Producing NIDA Opportunities (C3PNO). C3PNO is the coordinating center for nine National Institute on Drug Abuse (NIDA) cohorts, and focuses on the linking methods based on IRT to allow for combining data across cohorts to address questions at the intersection of HIV and substance use [35]. Depression is a key health outcome variable across most C3PNO cohorts, given its prevalence among people living with HIV (PLWH) and persons at risk of HIV [36], and the positive association between depression and substance use [37]. The cohorts participating in C3PNO, however, vary in terms of population characteristics, as well as assessment instruments. To facilitate cross-cohort data harmonization, Schalet et al [38] conducted a linking study of CES-D, PROMIS Depression, and PHQ-8 instruments, coupled with DIF analysis to compare item response characteristics in a general population sample versus C3PNO cohorts.

Establishing a new linking relationship–between the BSI and PROMIS Depression–would enable additional harmonization across C3PNO cohorts. BSI is a general psychological tool to assess patients at intake for psychological problems [9]. PROMIS is a PRO system of instruments supported by the National Institutes of Health (NIH) beginning in 2004. PROMIS instruments measure domains of overall well-being, such as physical, mental, and social health across many diseases [5, 39]. The PROMIS Depression scale has been validated and linked to other legacy measures in some studies [16, 40].

These two PRO instruments–BSI and PROMIS Depression–were previously linked to enable longitudinal analysis in the RADAR study [33, 41, 42]. Kaat et al. collected data from a sample of men who have sex with men (MSM) with a wide age range and multiple demographic characteristics to facilitate this linking. Another C3PNO cohort, the Healthy Young Men’s (HYM) cohort, also administered both the BSI and the PROMIS Depression scales at a single time point, enabling a new linking analysis. The HYM study is a longitudinal study that examines the individual, familial, interpersonal, and community factors impacting drug use, HIV risk-related behaviors, and engagement in HIV care among young MSM [43, 44].

Given the difference across the two samples, the present study examines whether the linking relationship between the BSI Depression subscale and the PROMIS Depression scale computed from the RADAR sample performs similarly to the new linking relationship we derived from the HYM sample. By doing so, this analysis could serve as a template for replicating linking analysis and provides practical guidelines for BSI-PROMIS crosswalks in similar samples.

## Methods

### Participants

The data of this study was collected from 448 MSM in the HYM cohort with the approval from the Children’s Hospital Los Angeles Institutional Review Board. Informed written consent was obtained from all participants. 80% of the sample were Hispanic or Black/African-American, and the age range was between 16 and 25 years old. Around 11% were diagnosed with HIV positive. Table 1 contrasts the HYM demographic characteristics with that from the previous linking study conducted by Kaat et al based on the RADAR study. Although both samples were comprised of MSM, they differed in age, race, and ethnicity. As shown in Table 1, the HYM sample was younger and included a higher proportion of Hispanic/Latinx and Black/African-American participants.

**Table 1:** Demographics of the HYM sample and the Kaat et al. sample.

Variables	HYM	Kaat et al*
Sample Size	448	2009
HIV positive (%)	11.3%	15.4%
Age (range, mean; yrs)	[16,25], 22.3	[18,76], 35.3
Race and Ethnicity (%)		
Hispanic	59%	18%
Black/African-American	21%	8%
White/Caucasian	11%	60%
Mixed or other	9%	14%

Note.

*The demographics of the Kaat et al. sample were extracted from the Kaat et al. [33].

### Measures

The BSI instrument is designed to assess psychological problems with an overview of participants’ symptoms and their severity [9, 45]. It is composed of three composite Global Indices and nine Symptom Scales, one of which is the Depression subscale. The Depression subscale was analyzed in the current study. Participants rated the extent to which they have been bothered (0 = "not at all" to 4 = "extremely") in the past week on six depressive symptoms. The total score was the sum of the six item scores, with higher scores indicating higher depression level.

The PROMIS Depression bank v1.0 for adults consists of 28 items assessing the negative emotion and cognition symptoms with a 7-day time frame [6]. Items were developed using both qualitative and quantitative methods [6] and have been validated across diverse clinical samples [40]. It is based on a 5-point Likert scale with response options ranging from “Never” to “Always” and with higher scores indicating higher depression level. Item responses are analyzed and scale scores are estimated based on IRT. The scale scores are standardized as T scores (Mean = 50; standard deviation [SD] = 10) based on a general population [5, 39]. Both BSI and 23 items from the PROMIS Depression item bank were administered to HYM cohort participants. Among the 23 PROMIS items, five items showed DIF between HYM and the general population [38]. The rest 18 items were analyzed in this linking study.

In the study of Kaat et al., an 8-item short form (PROMIS Depression 8a) from the adult PROMIS Depression v1.0 item bank was used. To make a fair comparison between the two resulting crosswalks, we also analyzed items of this short form extracted from the 18-item set.

### Fixed parameter calibration

The Kaat et al. study applied and compared the equipercentile and IRT-based linking approaches and selected the fixed parameter calibration approach as optimal to compute the crosswalk. To be consistent with the Kaat et al. study, we applied the same method for the linking analysis based on the HYM sample: IRT-based fixed parameter calibration. In the linking process, the item parameters of the anchor scale (the PROMIS Depression scale) were fixed at their established item parameters calibrated based on US general population. The item parameters of the legacy measure (the BSI Depression scale) were then calibrated based on the metric of the established item parameters of the anchor measure. Consistent with recommendations for fixed parameter calibration, we freely estimated and updated the latent distribution while calibrating the linked items, which has been referred to as multiple weights updating and multiple EM cycles [46]. By this process, the scores of the legacy measure items were estimated on the PROMIS metric and can be converted to T scores that would be aligned with PROMIS measures. We then used the Lord and Wingersky recursive algorithm [26] for EAP summed scoring to compute the crosswalk based on the linked item parameters of the BSI scale, which represents the most probable T score associated with each raw summed score [47].

### Data analysis

The HYM study adopted a single-group linking design, in which each participant received the two measures at the same visit. Before conducting the linking analysis, we checked the linking assumptions of similarity in content measured by the two measures and unidimensionality. First, to verify the similar content assumption, the item content of each measure was first qualitatively inspected and compared. Second, the disattenuated Pearson’s correlation between the raw scores of the two measures was calculated to determine whether they measure the similar construct. Third, confirmatory factor analysis (CFA) and bi-factor exploratory factor analysis (bEFA) were used to assess the unidimensionality of the combined scale (i.e., BSI Depression and PROMIS Depression *8a*). The following model fit criteria were used to evaluate the combined scale’s relative unidimensionality: RMSEA ≤ 0.08, CFI ≥ 0.95, TLI ≥ 0.95 [48–50]. An Omega Hierarchical (OmegaH) statistic [51, 52] ≥ 0.70 [53] also speaks to the broad unidimensionality of the combined scale. We investigated all quantitative linking assumptions using the psych package in R [54]. Data for this study are not publicly available. The analysis code for this study are available by emailing the corresponding author.

After the above assumptions have been checked, we implemented the fixed parameter calibration method using the PROsetta R package [55] and conducted a linking analysis between the BSI Depression subscale and the PROMIS Depression 8a and the PROMIS Depression 18-item set, respectively. We first compared the two sets of BSI Depression item parameter computed by linking the BSI Depression subscale to the short form and the 18-item set. Then, we evaluated the differences compared to the item parameters from the Kaat et al. study by plotting the slope and threshold parameters with their 95% confidence intervals for both samples. We then plotted the crosswalks and the interval indicating one standard error of measurement (SEM) above or below each score computed from the two samples to evaluate if the two crosswalks are similar. SEM explains how much the measurement error may spread out around each score. Finally, to evaluate the validity of the crosswalk table in the Kaat et al. study, the *crosswalk-derived* T score was compared with the *observed* T score using Pearson product-moment correlations and the mean, SD, RMSD of score differences. We also presented Bland-Altman plots and calculated intraclass correlation (ICC) coefficients to demonstrate the agreement between the observed and the crosswalk derived T scores.

## Results

### Assumptions

We examined the item content of the two measures and confirmed that both instruments mainly measure depressive symptoms. In addition, the disattenuated Pearson’s correlation between the BSI Depression scores and the PROMIS Depression 8a raw summed scores was high (*r* = 0.82), which further supported the assumption that the two measures assess a similar health outcome. Regarding unidimensionality, the model fit indices of a single-factor CFA model suggested an adequate unidimensional data-model fit (RMSEA = 0.082, CFI = 0.980, TLI = 0.977). The OmegaH (= 0.84) statistic from the bEFA analysis suggested that the combined scale was sufficiently unidimensional. For the linking between the BSI Depression subscale and the PROMIS 18-item set, these assumption analyses showed similar results.

### Descriptive statistics

The summary statistics for the scores of the two measures are shown in Table 2. The summary statistics for the T scores of the two samples showed that the HYM sample had a lower average depression level than the sample analyzed in the Kaat et al. study with a similar standard deviation and interquartile score range. Consistently, the average BSI raw summed score of the HYM sample was lower than the Kaat et al. sample. The effect size of the mean difference was 0.31 for PROMIS T scores and 0.47 for BSI scores.

**Table 2:** Summary statistics for the scores of the BSI Depression and the PROMIS depression.

	Kaat et al.		HYM	
	T score	BSI raw score	T score	BSI raw score
Mean	54.4	6.1	51.5	4.0
Median	53.5	4.0	51.7	3.0
SD	9.6	5.8	9.3	4.1
Minimum	38.2	0	38.2	0
Maximum	81.1	24	81.1	24
Interquartile range	[48.3, 60.7]	[2, 9]	[45.0, 57.7]	[1, 6]

### Item parameters

Following the analysis plan, we linked the BSI Depression subscale to both the PROMIS Depression 8a short form and the PROMIS Depression 18-item set. After fixing the item parameters of the two PROMIS Depression scales, two sets of item parameters for the BSI Depression subscale were computed (Table 3). They were similar with differences within 0.1, suggesting the consistency in the linking results with either short form or 18-item set being used as the anchor measure. This finding was also supported by the similar means and SDs of the T scores between the short form and the 18-item set (M_18-item set_ = 51.98, SD_18-item set_ = 9.12; M_SF_ = 51.53, SD_SF_ = 9.27).

**Table 3:** Transformed IRT-based item parameters based on the linking of the BSI depression and the PROMIS depression 8a and the PROMIS depression 18-item set.

	Item	a	b1	b2	b3	b4
8a	BSI-D 1	1.47	-0.02	1.33	2.31	3.32
	BSI-D 2	1.99	-0.29	0.82	1.45	2.33
	BSI-D 3	2.38	0.10	1.15	2.00	2.73
	BSI-D 4	3.70	0.79	1.51	1.85	2.54
	BSI-D 5	2.68	0.55	1.43	1.97	2.48
	BSI-D 6	2.41	1.78	2.48	2.72	3.06
18-item set	BSI-D 1	1.48	0.01	1.34	2.31	3.31
	BSI-D 2	2.08	-0.24	0.82	1.43	2.29
	BSI-D 3	2.42	0.12	1.14	1.98	2.71
	BSI-D 4	3.78	0.79	1.51	1.84	2.54
	BSI-D 5	2.67	0.56	1.44	1.98	2.49
	BSI-D 6	2.31	1.82	2.54	2.79	3.14

*Note*. a’s denote the slope parameters. b’s denote the threshold parameters.

**Fig 1:**
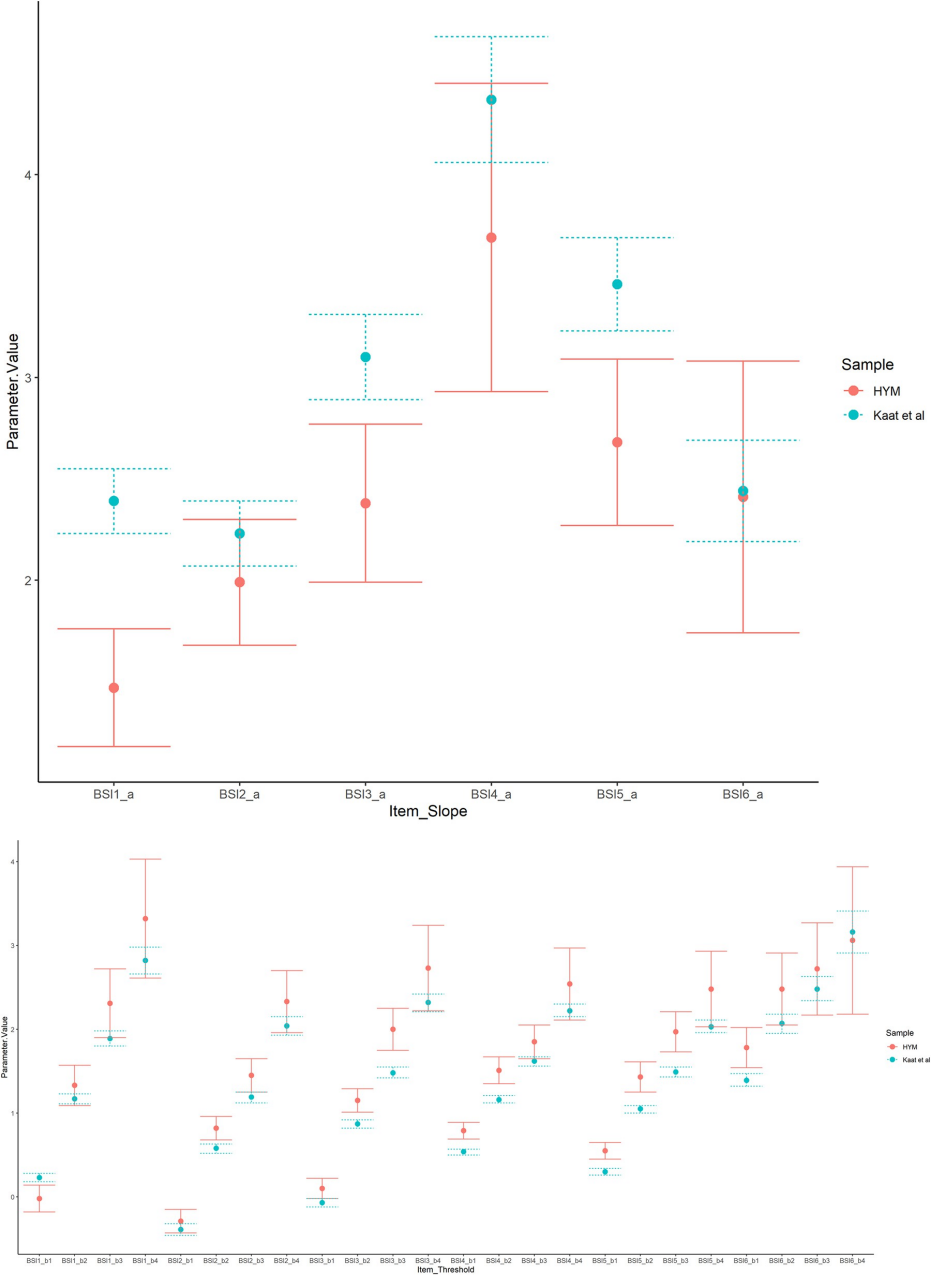
Item parameters and confidence intervals of the two studies. *Note*. Fig 1a: the slope parameters. Fig1b: the threshold parameters.

We also compared the item parameters of the BSI Depression subscale computed based on the HYM sample to those published in the Kaat et al. study. To allow for a fair comparison, we used the item parameters of the short form based on the HYM sample in the comparison. The two sets of item parameters were found to be different, which was expected when two different samples were analyzed for a linking analysis of the same PRO instruments. To evaluate how different they were, we plotted each parameter of the two sets with its 95% confidence interval (Fig 1). In general, the HYM sample showed wider confidence intervals for all the item parameters due to its smaller sample size. The slope parameters of the HYM sample were smaller than the ones estimated in the Kaat et al. study, suggesting that the BSI Depression items were less discriminating in the HYM sample. After taking into account the confidence intervals, the slope parameters of three items overlapped, suggesting the discrepancies were small. However, those of the other three items did not overlap. These three items are “Feeling no interest in things”, “Feeling blue”, “Feeling hopeless about the future”. Examining the frequency of each response option for these three items in the two samples, we found that participants of the HYM study tended to concentrate their responses on “Not at all” or “A little bit”, while participants of the Kaat et al. study had a higher proportion of responses endorsing “Moderately”, “Quite a bit”, “Extremely” than the HYM sample. This explains the disparity in the slope parameters of these three items between the two linking analyses.

Regarding the threshold parameters, fourteen out of twenty-four parameters showed overlapping confidence intervals between the two samples, suggesting there was not enough evidence to conclude that a replicable difference between these threshold parameters was found. However, ten parameters did not overlap in confidence intervals and the HYM sample tended to have higher thresholds than those in the Kaat et al. study. In particular, three thresholds of the item “Feeling hopeless about the future” were higher than that in the Kaat et al. sample. Moreover, the thresholds to reach high response options (i.e., “Quite a bit”, “Extremely”) across all six items showed larger difference between the two samples than low response options. Higher thresholds suggest a higher depression level is needed to reach the category threshold, indicating that respondents who endorsed high response options in the HYM sample had more severe depression level than those in the Kaat et al. sample.

### Crosswalks

**Fig 2:**
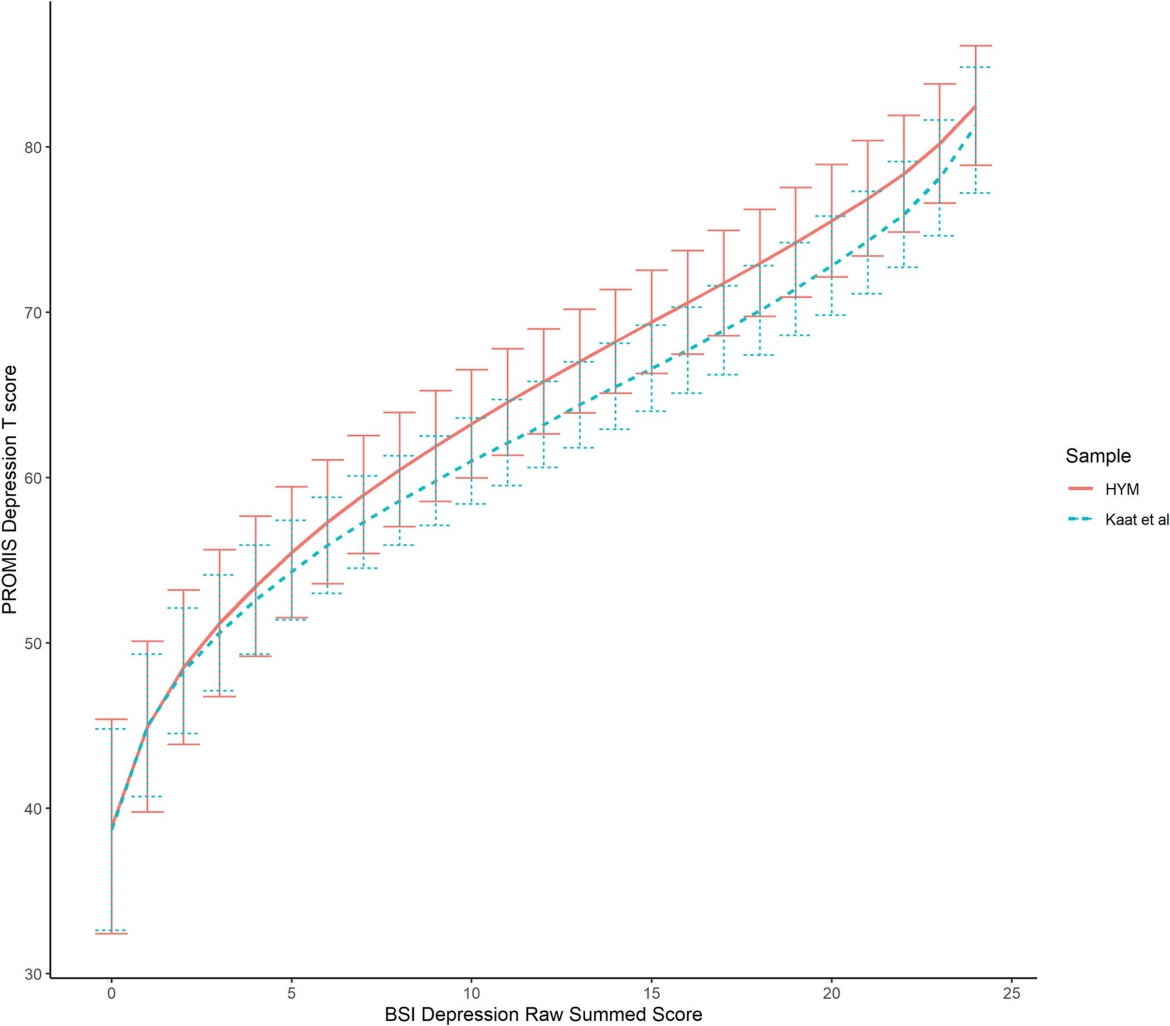
The crosswalks computed from the HYM sample and the Kaat et al. sample.

We plotted the crosswalk tables computed from the fixed parameter calibration method for both samples (Fig 2). In this plot, we showed the SEM above and below each score. Each curve depicts the linking relationship between the BSI Depression subscale and the PROMIS Depression 8a. Although the two linking curves showed the similar shape and trend, they varied from each other in a gradual way: larger variation for higher scores and smaller at two extreme ends. The crosswalk computed from the HYM sample tended to covert the BSI Depression score with a higher PROMIS Depression T score than the Kaat et al. sample. It is consistent with the abovementioned finding of higher thresholds at high categories in the HYM sample. All differences between the two crosswalks were within three T score points, and well within each score’s SEM. When both crosswalks were applied to the HYM data, the mean difference of the two sets of crosswalk-derived scores was −0.82. Moreover, the SEM intervals overlapped between the two samples, speaking to the consistency of the two crosswalks.

We further evaluated the validity of the Kaat et al. crosswalk in recovering the PROMIS Depression 8a T score and the 18-item set T score (Table 4). As expected, the crosswalk computed from the HYM sample showed lower mean difference between the observed and the crosswalk derived T scores than the crosswalk in the Kaat et al. study, but the SD of score difference was higher than that of the Kaat et al. study and the RMSD of score difference were very similar. We also presented Bland-Altman plots in S1 and S2 Figs and calculated intraclass correlation (ICC) coefficients to demonstrate the agreement between the observed and the crosswalk derived T scores in the PROMIS Depression 8a T score and the 18-item set across the full range of T scores. The shape of the Bland-Altman plot shows a floor effect in the samples, which suggests limited comparisons across groups for individuals who are not exhibiting depressive symptoms, but the linking bias—that is, the mean difference between methods—is small. The ICC values were 0.80 for both measures, indicating acceptable agreement between the two score. The mean differences of both the short form and the 18-item set observed T score versus the Kaat et al. crosswalk derived T score were 1.25 and 1.69. However, as Fig 2 shows, the SEM interval of the HYM sample overlaid on that of the Kaat et al. sample. As a result, the crosswalk in the Kaat et al. study can appropriately recover both the short form and 18-item set observed T scores. To further evaluate the impact of age on the crosswalk, we extracted data of participants from the Kaat et al. sample with a similar age range (i.e., [18, 25]) as in the HYM sample and found negligible differences (< 1 T-score point) between the Kaat et al. crosswalk based on the full sample and the crosswalk computed for the young MSM in the Kaat et al. sample. This finding further supported the validity of the Kaat et al. crosswalk.

**Table 4:** The difference between the observed and crosswalk-derived T scores in the HYM sample.

Linking	Method	Correlation	Mean*	SD*	RMSD*
PROMIS Depression 8a	HYM crosswalk	0.79	0.44	5.92	5.92
	Kaat et al. crosswalk	0.78	1.25	5.80	5.93
PROMIS Depression 18-item set	HYM crosswalk	0.80	0.73	5.62	5.67
	Kaat et al. crosswalk	0.80	1.69	5.51	5.76

*Note*.

*The mean, SD and RMSD of the differences between observed versus crosswalk-derived PROMIS T Scores.

## Discussion

Given the prevalence of depression among adults [56], it becomes essential to identify efficient assessment tools and representative data for the analysis of depression, and psychometric methods that can aggregate information and contribute to interventions that can be specific to populations of all groups. Linking of various measures and data harmonization can facilitate the examination of depression as a generic mental health construct over time and comparisons across multiple samples. Although population invariance is theoretically assumed in the linking analysis, we must be cautious before using a crosswalk table to convert scores for a sample that is different from the linking sample. This concern provides an impetus for validating crosswalks across multiple samples to make them generalizable to a broad spectrum of individuals, and making specific recommendations on their usage in terms of target population. The current study makes a practical contribution to this issue by replicating the linking analysis of two measures on an independent sample, provisionally validating the crosswalk table of the Kaat et al. study, and serving as a template for subsequent linking studies to validate their crosswalks.

The current study first presented the similarities and discrepancies between the item parameters of the BSI Depression subscale computed from the two samples. In short, the slope parameters of the Kaat et al. study were more discriminating than the HYM sample, while the threshold parameters of the HYM sample requires a more severe depression level to reach higher response categories than the Kaat et al. sample. Such discrepancies might be explained by the differences in sample characteristics; and such different BSI item parameters computed based on two samples may result in a different linking relationship and crosswalk, which need to be further verified. Second, the discrepancy between the two crosswalks was smaller than three T-score points across the full score range, a threshold previously considered to be small in the context of linking [57]. As shown in Fig 2, the SEM of each crosswalk score point overlapped. Moreover, the Kaat et al. crosswalk generated linked scores that were close to the observed T scores of both the PROMIS Depression 8a short form and the PROMIS Depression 18-item set in the HYM sample, such that the mean score difference was less than two T-score points. Given the smaller sample size of HYM compared to the sample of Kaat et al. (N_1_ = 448 vs N_2_ = 2009), we conclude that there is not enough evidence to recommend the usage of the HYM-based crosswalk over the Kaat et al. crosswalk. Therefore, we encourage the continued use of the Kaat et al. crosswalk to convert the BSI Depression subscale to the PROMIS Depression T score in similar samples.

In the current literature on linking two health outcome measures, few studies have evaluated the external validity of their crosswalks. As an example, one study linked legacy pain interference measures with the PROMIS pain interference scale, and computed a crosswalk for the individuals with multiple sclerosis and the general population, respectively [34]. They found the difference between the two crosswalks was very small. Two other studies incorporated cross-validation in the linking design [57, 58]. However, most linking studies did not undertake the comparison of crosswalks computed by different samples, probably due to a lack of data from a separate sample.

This study provides a blueprint and recommendation for future studies to compare linking results across studies. Specifically, it replicates the IRT-based linking method and compares the pairs of item parameters and the associated SEM from both studies (Figs 1 and 2). When the majority of paired parameters are outside the SEM range of both estimates, it would suggest a separate linking analysis might be needed for each group. In addition, we compared the crosswalk tables to each other: when the score SEM no longer overlap, this would tell researchers to use separate crosswalks. We also evaluated how close the T scores generated by the previously stablished crosswalk were to the observed T scores in the new sample. These agreement statistics, such as the mean bias, serve as an aggregate difference; using this measure, group mean differences larger than a small effect size might be cause for concern.

Our study has a few limitations, which imply directions for future studies. First, the sample size we used to validate the established crosswalk was not very large (N = 448). Future studies may use a larger sample to examine the external validity of an established crosswalk. Second, although the two crosswalks were similar, there were some relatively large differences in the slope parameters and the location parameters at higher score levels. One reason for such discrepancy might be due to the relatively small number of participants. Another reason might be the differences in sample characteristics: as we noted, although both samples were MSM participants, the HYM sample was younger and included a higher proportion of Hispanic/Latinx and Black/African-American participants, and far fewer White/Caucasian compared to the Kaat et al. sample. It is possible that non-white or younger participants respond differently to some depression questions but not others, given that depression is found to be less prevalent among older adults than among younger adults [59]. Additionally, the study sample did not include female participants. Future research, with larger aggregated datasets, could address these questions with a differential item functioning analysis regarding race, age, gender and other relevant factors. We also recommend subsequent researchers to validate this crosswalk table on samples and subpopulations that are different from the current study sample.

Finally, this study focuses on the IRT-based fixed parameter calibration approach as the established crosswalk was computed using this approach. Although a number of previous studies have shown convergence across multiple linking methods in a range of PRO constructs, future studies may evaluate and compare the validity of the crosswalk computed by different linking methods including IRT-based or equipercentile approaches. It is worth understanding whether each linking method can generate crosswalks that are robust to different sample sizes. Moreover, due to the disattenuated Pearson’s correlation of 0.82 lower than the 0.866 threshold recommended by Dorans et al [17], we caution users that the crosswalk of this study may be used for group level comparisons but not for individual level clinical decision making [15].

In conclusion, this study applied a series of validation steps to determine if an established crosswalk for the conversion between the BSI Depression subscale scores and the PROMIS Depression T scores is valid and replicable for an independent sample. In our analysis, some item parameters of the BSI measures derived from the two samples were different. However, the discrepancy between the crosswalks computed from the two samples was well within each crosswalk score’s SEM range. In addition, the established crosswalk can provide linked scores that are adequately similar to the observed scale scores in the validation sample. Hence, this study verifies the reproducibility of the established crosswalk in an independent sample. Future linking studies can evaluate the external validity of this crosswalk to other populations.

## Supporting information

S1 Fig Bland-Altman plot for observed and IRT fixed parameter calibration crosswalk linked scores from PROMIS depression item bank. (TIF)

S2 Fig Bland-Altman plot for observed and IRT fixed parameter calibration crosswalk linked scores from PROMIS depression short form 8a. (TIF)
