# Supplementary figures and images for: Can a linking crosswalk table be applied to a different population? An independent validation study for a crosswalk between BSI depression and PROMIS depression scales
Source: PLoS One. 2022 Nov 28;17(11):e0278232. doi: 10.1371/journal.pone.0278232 (PMC9704687; doi:10.1371/journal.pone.0278232)

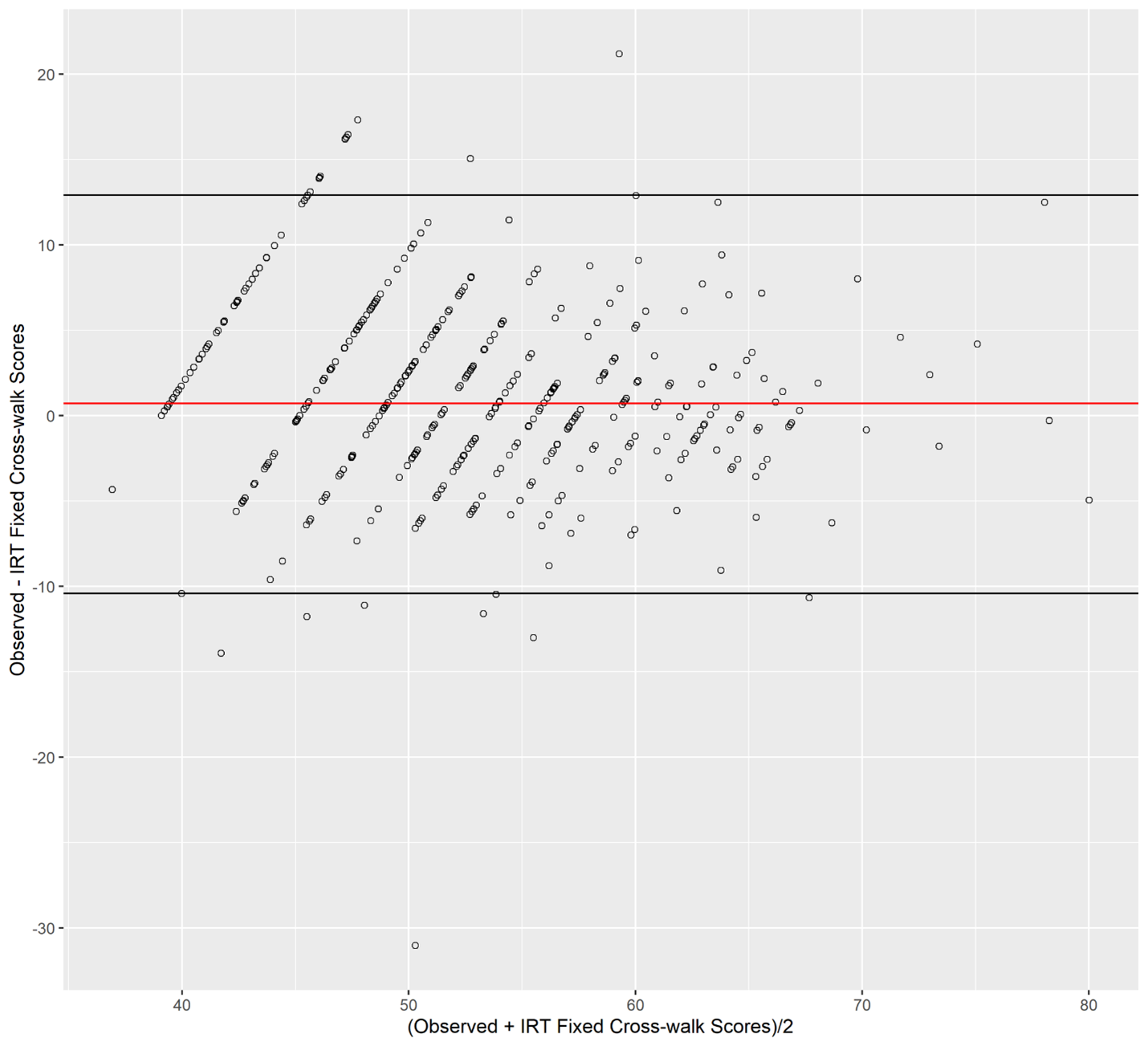

Supplement: S1 Fig — (TIF) [file pone.0278232.s001.tif]

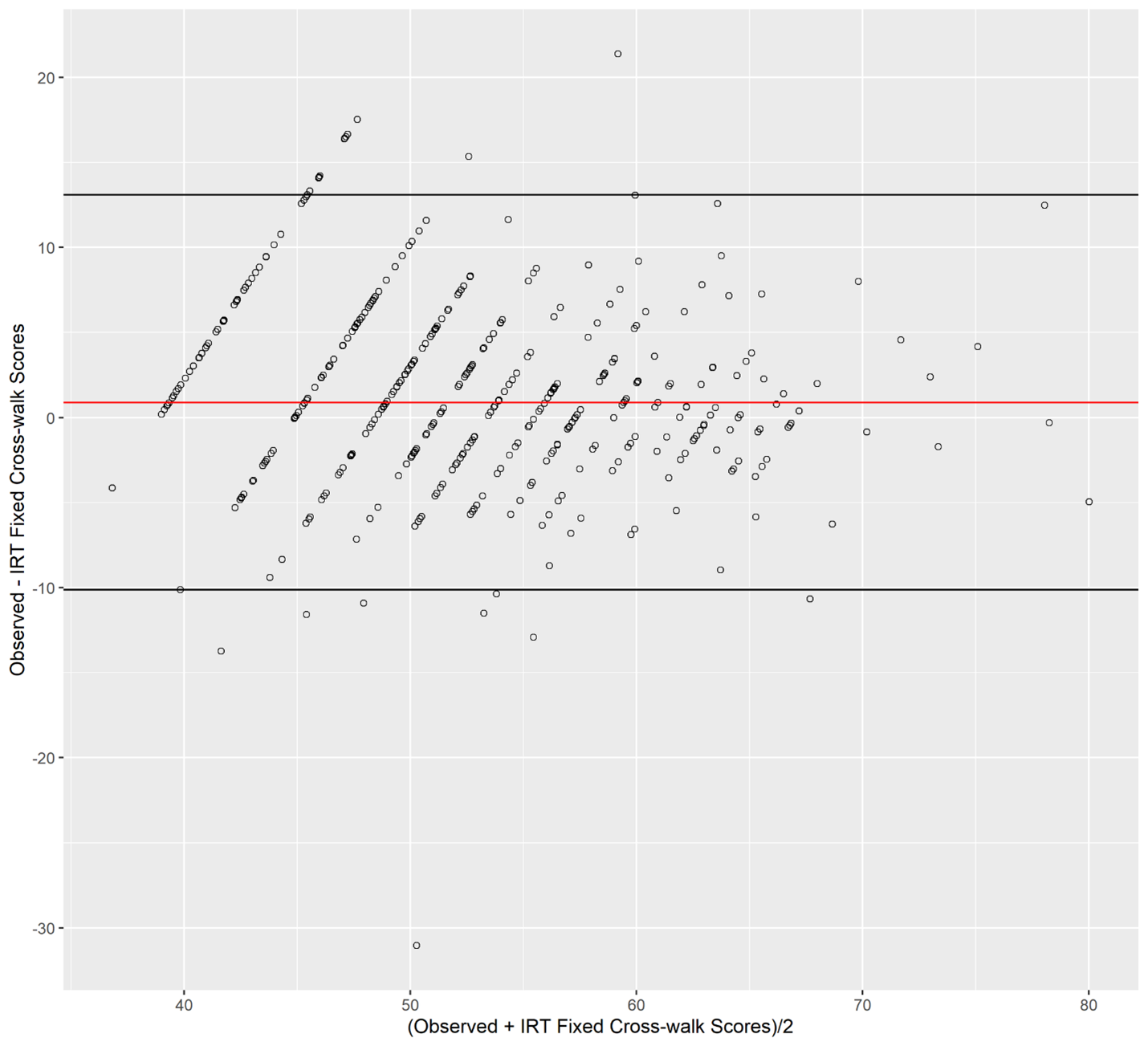

Supplement: S2 Fig — (TIF) [file pone.0278232.s002.tif]
